# Prognostic value of mitochondrial CKMT2 in Pan-cancer and its tumor immune correlation analysis

**DOI:** 10.1038/s41598-023-46468-3

**Published:** 2024-01-03

**Authors:** Wei Lin, Jiamin Zhou, Yili Ma, Liuxing Ge, Yiling Luo, Yaobin Wang, Sufang Zhou

**Affiliations:** 1 State Key Laboratory of Targeting Oncology, National Center for International Research of Bio-targeting Theranostics, Guangxi Key Laboratory of Bio-targeting Theranostics, Collaborative Innovation Center for Targeting Tumor Diagnosis and Therapy, Guangxi Medical University, Nanning, 530021 Guangxi China; 2 Department of Biochemistry and Molecular Biology, School of Basic Medical Sciences, Guangxi Medical University, Nanning, China; 3 Department of Pathology, Affiliated Cancer Hospital of Guangxi Medical University, Nanning, China

**Keywords:** Cancer, Computational biology and bioinformatics, Immunology, Molecular biology, Medical research, Oncology

## Abstract

Mitochondrial metabolism has been shown to play a key role in immune cell survival and function, but mitochondrial creatine kinase 2 (CKMT2) has been relatively little studied about tumor immunity. We aimed to explore the prognostic value of CKMT2 in 33 cancer types and investigate its potential immune function. We used a range of bioinformatics approaches to explore the potential carcinogenic role of CKMT2 in multiple cancers. CKMT2 was lowly expressed in 14 tumor tissues and highly expressed in 4 tumor tissues. Immunohistochemical assays showed overexpression of CKMT2 in colon cancer and rectal cancer. CKMT2 overexpression was positively correlated with the prognosis of lung adenocarcinoma and prostate cancer. CKMT2 overexpression is mainly enriched in the adaptive immune system and immune regulatory pathways of immunoglobulins. Seven cancers were positively correlated with low CKMT2 expression in tumor microenvironment analysis. Among the five cancers, low expression of CKMT2 resulted in better immunotherapy treatment outcomes. There was a strong correlation between CKMT2 and most immune-related genes in specific cancer types. CKMT2 plays an important role in tumorigenesis and cancer immunity and can be used as a prognostic biomarker and potential target for cancer immunotherapy.

## Introduction

In recent years, cancer immunotherapy has become a prominent cancer treatment, especially immune checkpoint blockade therapy^1^. With the continuous development and improvement of public databases such as The Cancer Genome Atlas (TCGA), it is possible to identify new immunotherapeutic targets by performing pan-cancer expression analysis of genes and evaluating their correlation with clinical prognosis and related signaling pathways^2^.

Mitochondrial metabolism is now a key target for cancer therapy, and recent studies have shown that metformin exerts its anticancer effects by inhibiting mitochondrial ETC complex I^3^. Mitochondrial metabolism is required for cancer cell proliferation and tumorigenesis^4^. Therefore, a rational cancer treatment strategy should include the simultaneous targeting of multiple metabolic pathways or the targeting of specific metabolic pathways in combination with therapies that target oncogenic or signaling pathways. Mitochondrial metabolism has been shown to play a key role in immune cell survival and function, and inhibition of mitochondrial ETC suppresses the proliferation of effector T cells^5^ and downregulates regulatory T cell (Treg) function^6^. Understanding more about the tumor immune relevance of mitochondrial metabolism in regulating tumorigenesis will allow the design of combination therapies using mitochondrial metabolism inhibitors with other anticancer agents. Mitochondrial creatine kinase (CKMT2) is one of the creatine kinase (CK) isozymes that are tightly coupled to adenosine triphosphate (ATP) output via adenine nucleotide transport proteins or carriers, making it an important player in ATP synthesis and respiratory chain activity^7^. CKMT2 activity is associated with oxidative capacity as it increases the availability of ADP to the respiratory chain complex V, which regulates mitochondrial membrane potential (Δψm) and reactive oxygen species (ROS) formation^7,8^. Toll-like receptors (TLRs) TLR1, TLR2, and TLR4 can enhance ROS production by recruiting mitochondria into macrophage phagosomes and transferring tumor necrosis factor receptor-associated factor 6 (TRAF6) to mitochondria to participate in evolutionarily conserved signaling intermediates in the Toll pathway (ECSIT)^9,10^. This links innate immune signaling to the mitochondria, establishing them as the hub of innate immune signaling.

There are few references on the role of CKMT2 in cancer. CKMT2, also known as s-MtCK, belongs to the creatine kinase isoenzyme family. Overexpression of CKMT2 has been reported in malignant tumors^11^. This isozyme may represent a tumor marker^12^. Wang H et al. have shown that CKMT2 may be a key regulator involved in osteosarcoma formation^13^. High mitochondrial creatine kinase expression has been reported in hepatocellular carcinoma suggesting a poor prognosis and a highly malignant potential^14^. However, studies on the immune-related aspects of CKMT2 in tumors are still relatively few.

Long-stranded non-coding RNA (lncRNA) is a type of RNA defined as a transcript that is more than 200 nucleotides in length and is not translated into protein^15^. Growing evidence suggests that lncRNAs play complex and precise regulatory roles in cancer development and progression by acting as oncogenes or tumor suppressors^16,17^. They not only regulate the proliferation, differentiation, invasion, and metastasis of cancer cells but also the metabolic reprogramming of cancer cells^18,19^. Furthermore, lncRNAs play important functional roles in regulating the transcription and translation of metabolism-related genes. They act as decoys, scaffolds, competing endogenous RNAs (ceRNAs), etc. ultimately leading to metabolic reprogramming in cancer^20^. However, no potential lncRNAs associated with CKMT2 in cancer have been reported.

From the rapidly accumulating data in large-scale cancer genomics studies, many studies have focused on pan-cancer analysis to estimate genome-wide, frequently mutated genes and other common genomic features associated with cancer onset and progression^21,22^. In this study, we analyzed the specific role and potential mechanisms of CKMT2 in a pan-cancer dataset. On the one hand, we analyzed the relationship between CKMT2 expression and patient prognosis in 33 cancer types. In addition, we further evaluated the expression of CKMT2 and its association with tumor-infiltrating immune cells. Our findings reveal a possible role of CKMT2 in tumorigenesis and progression of multiple cancers, suggesting that CKMT2 is a potential prognostic and immunotherapeutic biomarker.

## Materials and methods

### Data collection and processing

CKMT2 gene expression data and clinical information from The Cancer Genome Atlas (TCGA) in tumor and corresponding normal samples were obtained using UCSC Xena (https://xena.ucsc.edu/ ) for exploring gene expression as well as clinical and phenotypic data. RNA sequencing data were examined for these tumor types after Log 2 transformation, as well as two-group t-tests; statistically significant differences were defined as * *p* < 0.05; ** *p* < 0.01; *** *p* < 0.001; **** *p* < 0.0001. R software (version 4.2.1) was used to analyze the data and the R package “ggpubr” was used to plot the box line graphs. Data from 10,327 TCGA tumor tissues and 730 TCGA normal tissues were analyzed. The gene expression profiling datasets GSE10950 (COAD = 24, Normal = 24), GSE37182 (COAD = 88, Normal = 84), GSE74602 (COAD = 30, Normal = 30), and GSE136735 (COAD = 6, Normal = 6) of colon cancer and normal tissues were obtained from GEO (https://www.ncbi.nlm.nih.gov/geo/database) . The GSE10950 dataset is based on the GPL6104 platform Illumina humanRef-8 v2.0 expression bead chip. The GSE37182 dataset is based on the GPL6947 platform Illumina HumanHT-12 V3.0 expression beadchip. GSE74602 dataset based on GPL6104 platform Illumina humanRef-8 v2.0 expression beadchip. GSE136735 dataset based on GPL16699 platform Agilent-039494 SurePrint G3 Human GE v2 8 × 60 K Microarray 039,381 (Feature Number version). *P* < 0.05 and |FC|> 2 were selected as screening conditions.

Full names of the tumors and corresponding abbreviations were given below: Adrenocortical Cancer (ACC); Bladder Cancer (BLCA); Breast Cancer (BRCA); Cervical Cancer (CESC); Bile Duct Cancer (CHOL); Colon Cancer (COAD); Large B-cell Lymphoma (DLBC); Esophageal Cancer (ESCA); Glioblastoma (GBM); Head and Neck Cancer (HNSC); Kidney Chromophobe (KICH); Kidney Clear Cell Carcinoma (KIRC); Kidney Papillary Cell Carcinoma (KIRP); Acute Myeloid Leukemia (LAML); Lower Grade Glioma (LGG); Liver Cancer (LIHC); Lung Adenocarcinoma (LUAD); Lung Cancer (LUSC); Mesothelioma (MESO); Ovarian Cancer (OV); Pancreatic Cancer (PAAD); Pheochromocytoma & Paraganglioma (PCPG); Prostate Cancer (PRAD); Rectal Cancer (READ); Sarcoma (SARC); Melanoma (SKCM); Stomach Cancer (STAD); Testicular Cancer (TGCT); Thyroid Cancer (THCA); Thymoma (THYM); Endometrioid Cancer (UCEC); Uterine Carcinosarcoma (UCS); Ocular melanomas (UVM).

### Genetic variation analysis

The cBioPortal database (www.cbioportal.org) was used to explore the analysis of genomic alterations in specific genes^23^. Select the “Pan-cancer analysis of whole genomes (ICGC/TCGA, Nature 2020)” dataset. Apply the “Cancer Types Summary” and “Cancer Type” buttons to visualize the frequency of CKMT2 copy number changes and mutations in 27 TCGA cancer types. The results are represented by the bar graphs drawn.

### Protein expression analysis

The Human Protein Atlas (HPA) (http://www.proteinatlas.org/) is a landmark protein research database containing protein expression from tumor and normal tissues for exploring CKMT2 expression at the protein level. IHC images of CKMT2 protein expression in 4 normal tissues including liver, kidney, colon, and myocardial tissues, and 4 tumor tissues including CHOL, COAD, PRAD, and BRCA were downloaded from HPA and analyzed. The antibody used for IHC is HPA051880.

### Immunohistochemical staining

Thirty-two paraffin specimens of colon and rectal cancer with complete clinical data from the Department of Pathology, Cancer Hospital of Guangxi Medical University were selected from January 2022 to December 2022. All patients underwent radical surgery for intestinal cancer, and none of them received radiotherapy or chemotherapy before surgery. The clinical information of the patients was as follows: female (n = 10), male (n = 22); age ≥ 60 (n = 5), age < 60 (n = 27); stage T1 (n = 3), T2 (n = 5), T3 (n = 24); and type of cancer were adenocarcinoma. Normal colonic and rectal tissues adjacent to the cancer were selected as controls (32 cases in total). This study was approved by the Ethics Committee of Guangxi Medical University (20,200,035). All patients provided informed consent. All experiments of this study were approved by the Ethics Committee of Guangxi Medical University and conformed to the ethical guidelines and regulations of Guangxi Medical University.

Immunohistochemical staining of CKMT2 was performed according to the following protocol. The formalin-fixed paraffin-embedded sections were dewaxed with methanol, repaired with antigen (heat-mediated in EDTA buffer, pH = 9.0), sealed for 30 min, incubated overnight with CKMT2 antibody (proteintech, 13,207–1-AP) at 4 °C at a dilution of 1:500. Then incubated with the second antibody at room temperature: HRP coupled with rabbit IgG. The sections were photographed by Olympus microscope (BX43). The positive area and staining intensity of CKMT2 in the sections were calculated by ImageJ plug-ins IHC Profiler^24^ and IHC Toolbox. Statistical analysis was carried out by GraphPad Prism 9, the positive areas of CKMT2 were tested by Wilcoxon, and the staining intensity of CKMT2 was tested by paired t-test. The significant difference in statistics is defined as * *p* < 0.05, ** *p* < 0.01 and *** *p* < 0.001, **** *P* < 0.0001.

### Cells and cell cultures

Human normal colon epithelial cells FHC and human colon cancer cells SW480 and RKO were kept in the laboratory of our group and cultured according to their guidelines. The culture fluid was changed every two days. RKO and FHC cells were cultured in RPMI1640 (Gibco, USA) supplemented with 10% fetal bovine serum (VivaCell, China), 100 U/mL penicillin, and 100 μg/mL streptomycin in a humidified atmosphere containing 5% CO_2_ at 37 °C. SW480 cells were cultured in Dulbecco’s modified Eagle medium (Gibco, USA) for culture.

### Quantitative real-time RT-PCR

RNA extraction was performed using the RNA Extraction Kit (Axygen). Complementary DNA was synthesized using the cDNA Reverse Transcription Kit (Genstar) according to the manufacturer’s instructions. RT-PCR was performed on StepOneTM and StepOnePlusTM Real-Time PCR systems using SYBR Selected Premix (Genstar). Primer sequence: CKMT2 homo-F: ACGAGGCTGGGAGTTCATGT, CKMT2 homo-R: AAAGCGTGGGTCCTTGCTG. GAPDH was used as an internal control and data were analyzed using the 2-ΔΔCt method.

### Survival prognosis analysis

To extract disease-specific survival (DSS), disease-free (DFI), and progression-free (PFI) survival information from TCGA and to reveal the relationship between CKMT2 expression and patient prognosis. The median CKMT2 expression in each tumor was used as a cutoff value to classify patients into high and low-expression groups. Survival data for each cancer type were assessed by the Kaplan–Meier survival method and log-rank test. Survival curves were plotted using the R packages “survminer” and “survival” and *p* < 0.05 was considered significant. In addition, univariate Cox models were used to assess the relationship between CKMT2. A risk ratio (HR) < 1 is considered to mean that CKMT2 is a protective factor for cancer; HR > 1 means that CKMT2 is a risk factor for cancer. Data were visualized as forest plots (using the “forestplot” R package).

### ceRNA network analysis

The ENCORI database (https://rnasysu.com/encori/) was used to explore possible target miRNAs for CKMT2 and predict target lncRNAs for hub miRNAs. We constructed the lncRNA-miRNA-mRNA ceRNA network using Cytoscape software (v3.10.1). The miRNA data of “Isoform Expression Quantification” in the TCGA-PRAD dataset were downloaded from the TCGA GDC (https://portal.gdc.cancer.gov/) database and analyzed.

### Gene set enrichment analysis

Correlation analysis of CKMT2 with all genes was performed using TCGA data. Genes associated with CKMT2 (*p* < 0.05) were selected for gene set enrichment analysis (GSEA). GSEA is executed using R packets “clusterProfiler”, “org.Hs.eg.db”, “enrichplot” and “limma” with the following parameters and selecting the “c5.all.v7.1.symbols.gmt” gene set.

### Immune-related gene co-expression, tumor microenvironment, and immune cell infiltration analysis

The association between CKMT2 expression and genes encoding MHC, immune activation, immune suppression, chemokines, and chemokine receptor proteins was analyzed using R software (version 4.2.1). Estimation of stromal and immune cells in malignant tissues using expression data (ESTIMATE) is a method for calculating stromal or immune scores, which represent the abundance of immune and stromal components, respectively. The higher the score, the greater the proportion of the corresponding component in the TME. CKMT2 expression Immune Score and Stromal Score for each cancer were obtained by “limma”, “preprocessCore” and “e1071” R packages and Spearman correlation analysis. Immunocytic infiltration correlation analysis was performed using R software (version 4.2.1) for data analysis. For each TCGA tumor type, patients were divided into two groups (high and low CKMT2 expression based on median CKMT2 expression levels) to compare the extent of immune cell infiltration. Calculate the Pearson correlation coefficient.

### Analysis of tumor mutational load and microsatellite instability

Somatic mutation data were downloaded from the UCSC XENA database for all TCGA patients, and TMB scores were calculated. Microsatellite instability (MSI) data from a study^25^. The relationship between CKMT2 expression and TMB or MSI was analyzed using Spearman’s correlation coefficient.

### Statistical analysis

T-test was used to estimate the difference in CKMT2 gene expression between cancer tissues and normal tissues. Survival analysis was analyzed by the Kaplan–Meier method and compared with the logarithmic rank test. The results were expressed by risk ratio, 95%CI, and *p*-value of the logarithmic rank test. Spearman’s or Pearson’s test was used to analyze the correlation between the two variables. The R software (version 4.2.1) and GraphPad Prism 9 were used for statistical analysis. *P* < 0.05 is considered to be statistically significant.

## Results

### The mRNA expression of CKMT2 is different in pan-cancer

**Figure 1:**
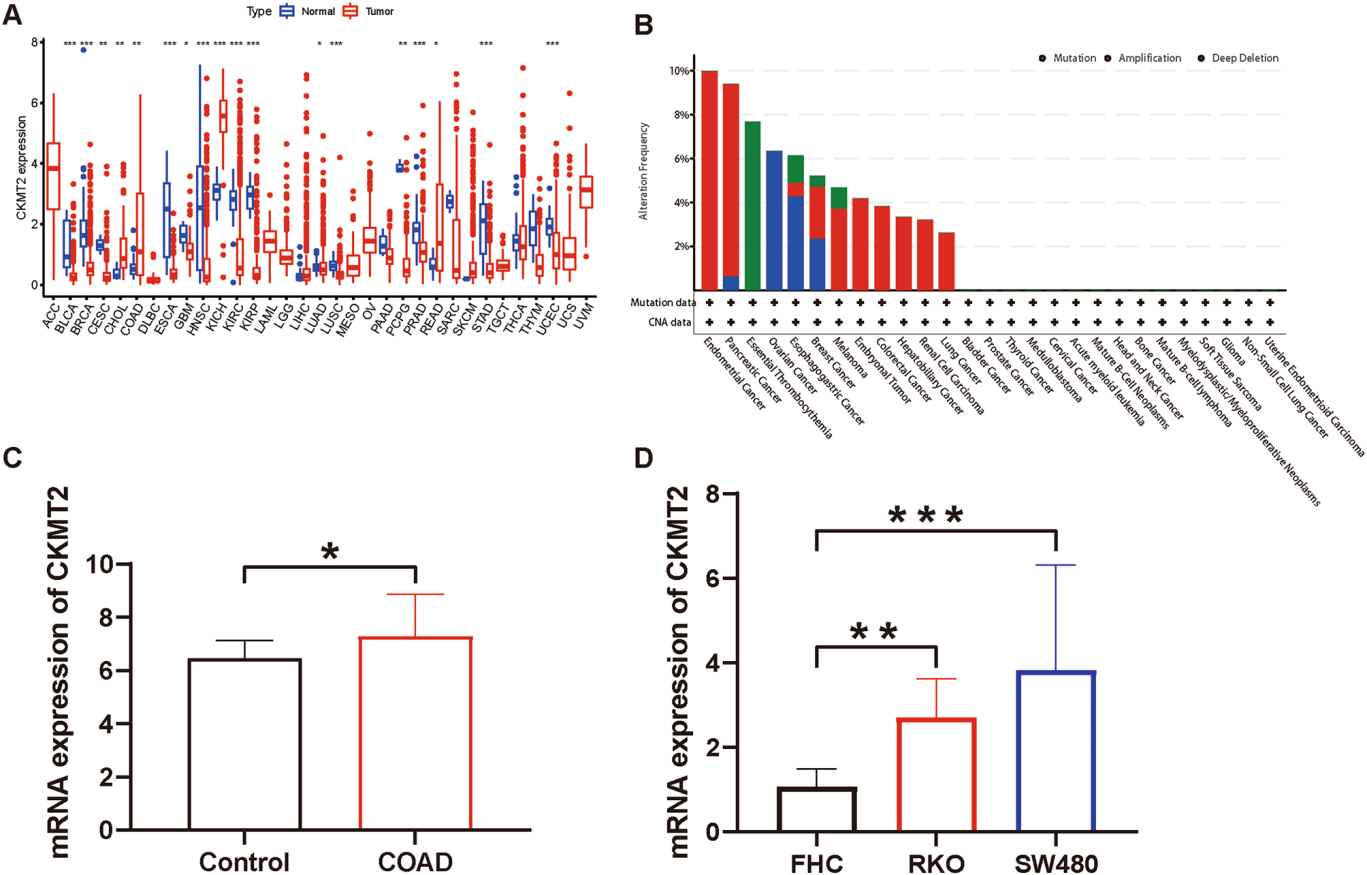
(**A**) CKMT2 mRNA expression levels in pan-carcinoma. CKMT2 expression differences between normal tissues and tumor tissues in the TCGA database. *p < 0.05, ** *p* < 0.01, *** *p* < 0.001, **** *p* < 0.0001. (**B**) Relationship between CKMT2 expression and gene changes. Type and frequency of CKMT2 genetic alterations in various cancers. (**C**) Relative mRNA expression of CKMT2 in Control (n = 148), COAD(n = 144) was based on the GEO database. (**D**) The mRNA expression level of CKMT2 was determined in two selected COAD cells by RT-qPCR and compared to FHC cells. The results of D were normalized to the GAPDH expression. Values are expressed as mean ± SD from three technical repeats. **P < 0.01, ***P < 0.001.

To compare the expression of CKMT2 between tumor and normal tissue, the difference in CKMT2 expression was analyzed with the data from the TCGA database. The results from the database show that CKMT2 is overexpressed in four of these cancers: CHOL, COAD, KICH, and READ. By contrast, low expression was observed in 14 cancers: BLCA, BRCA, CESC, ESCA, GBM, HNSC, KIRC, KIRP, LUAD, LUSC, PCPG, PRAD, STAD, UCEC (Fig. 1A). The cBioPortal database was used to evaluate the frequency of CKMT2 changes and mutation counts in cancer patients. Among all cancers, the frequency of CKMT2 change was the highest (10%) in patients with CESC, with "mutation and deep deletion" as the main type (Fig. 1B). The genetic changes in DNA are closely related to tumorigenesis and progression.

COAD was selected from four cancers overexpressing CKMT2 for CKMT2 gene expression analysis in the GEO database. From the analysis of GEO database data, it was known that CKMT2 was overexpressed in COAD (*P* < 0.05) (Fig. 1C). Human normal colon cells (FHC) and four human colon cancer cells (SW480, RKO) were also selected to detect the mRNA expression of the CKMT2 at the cellular level. The expression of CKMT2 was up-regulated (*P* < 0.05) in human colon cancer cells (SW480, RKO) compared to human normal colon cells (FHC) (Fig. 1D).

**Figure 2:**
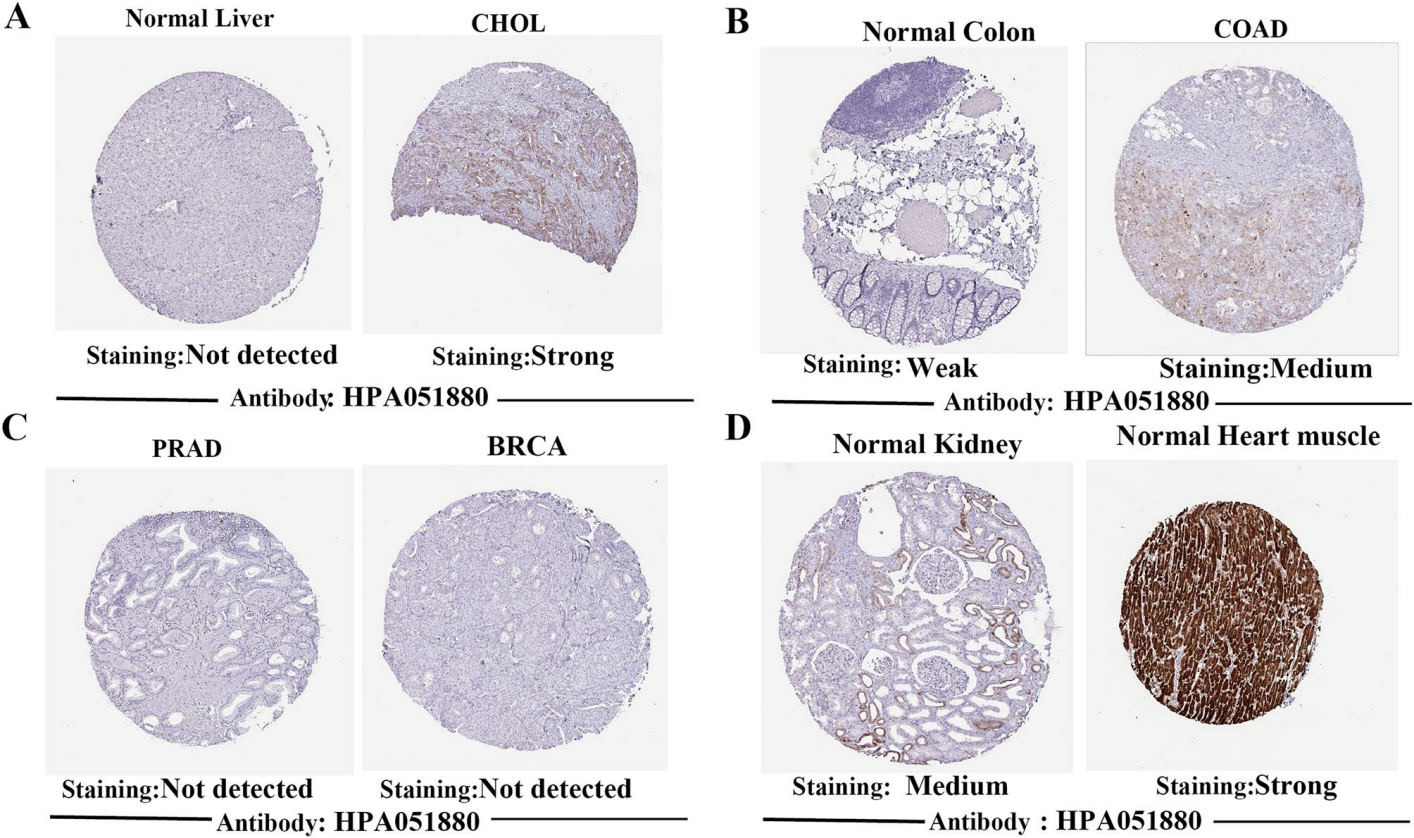
Immunohistochemical staining of normal and neoplastic tissues (IHC). (**A**) CHOL; (**B**) COAD; (**C**) PRAD and BRCA; (**D**) Normal renal tissue and Normal myocardial tissue.

At the same time, the relationship of CKMT2 mRNA expression in different pathological stages of various cancer types was evaluated, and it was found that the expression of CKMT2 in IV stage of BLCA, HNSC, LUAD, and THCA was lower than that in stage I. On the contrary, the expression of CKMT2 in stage IV was higher than that in stage I in KIRP (Supplementary Figure S1A-E). In addition, we further evaluated the expression of CKMT2 between normal and tumor tissues at the protein level from the HPA database. CKMT2 was expressed in normal colon, kidney, and myocardium, but not in normal liver tissue. It was not expressed in PRAD and BRCA but strongly expressed in COAD and CHOL (Fig. 2A–D). It can be concluded that CKMT2 protein expression is consistent with mRNA expression.

## Validation of CKMT2 overexpression in COAD and READ

**Figure 3:**
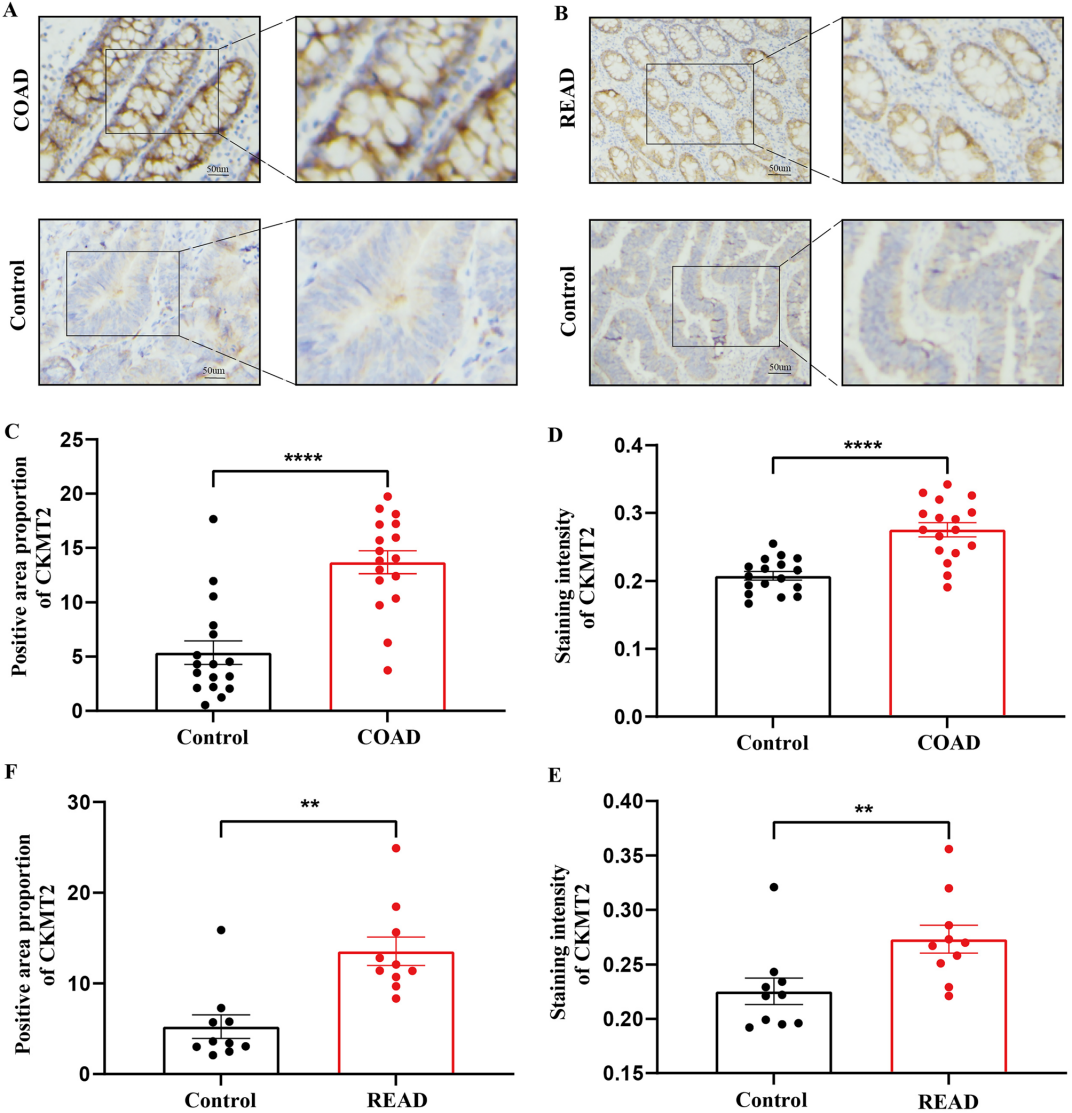
Protein expression of CKMT2 in COAD and READ. (**A**, **B**) Protein expression of CKMT2; (**C**, **D**) Positive area proportion of CKMT2 ;(**E**, **F**) Staining intensity of CKMT2. *p < 0.05, **p < 0.01, ***p < 0.001, ****p < 0.0001, *****p < 0.0001.

Twenty pairs of COAD and paracancerous specimens and 12 pairs of READ and paracancerous specimens were analyzed. It was found that CKMT2 was expressed in normal colon and rectal tissues and highly expressed in COAD and READ. Consistent with CKMT2 mRNA expression in COAD and READ, the results were corroborated and supplemented with the HPA database (Fig. 3A–F).

## CKMT2 has prognostic value

**Figure 4:**
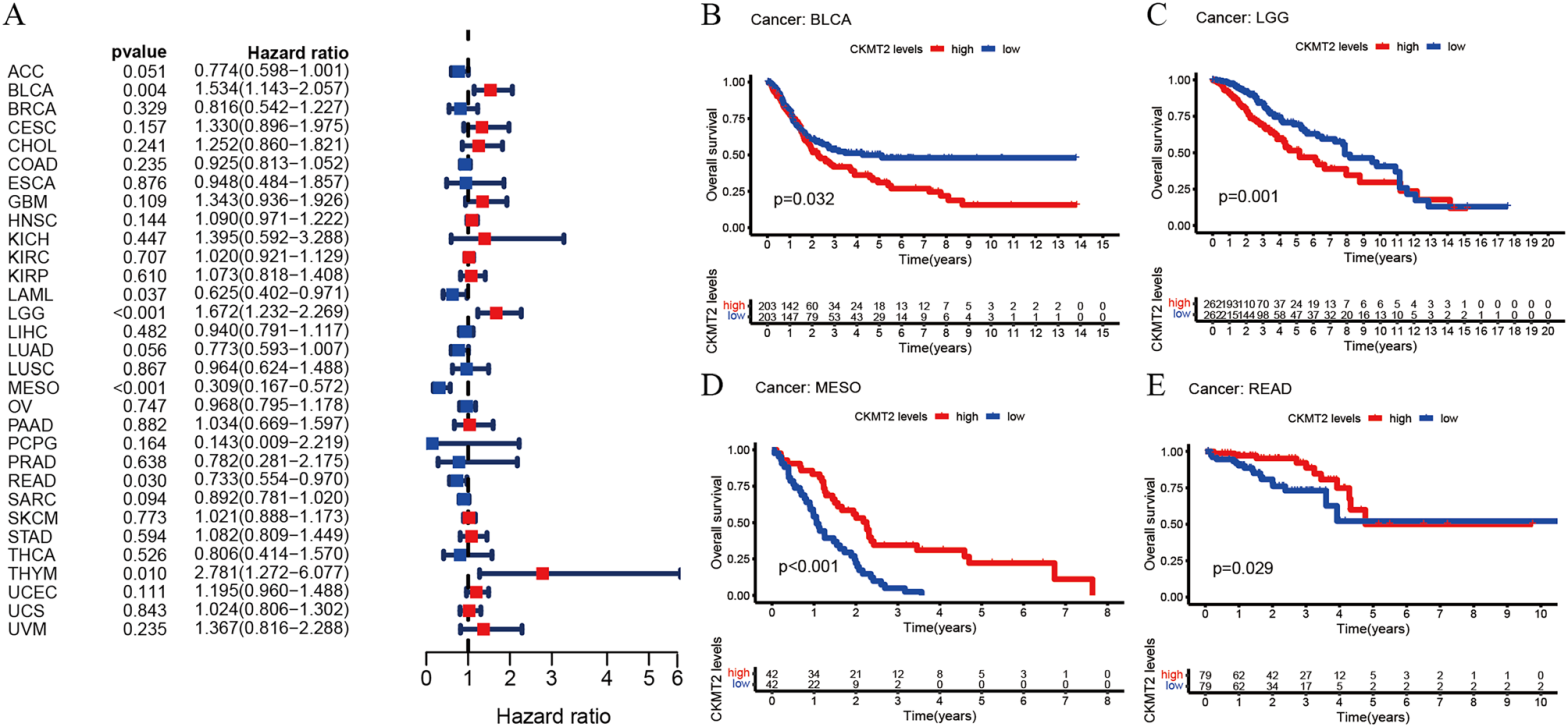
Relationship of CKMT2 expression with patient Overall Survival (OS). (**A**) The forest map shows the univariate Cox regression analysis results for CKMT2 in TCGA pan-cancer samples. (**B**–**E**) Kaplan–Meier OS curves of CKMT2 expression in the four most significantly associated tumors.

**Figure 5:**
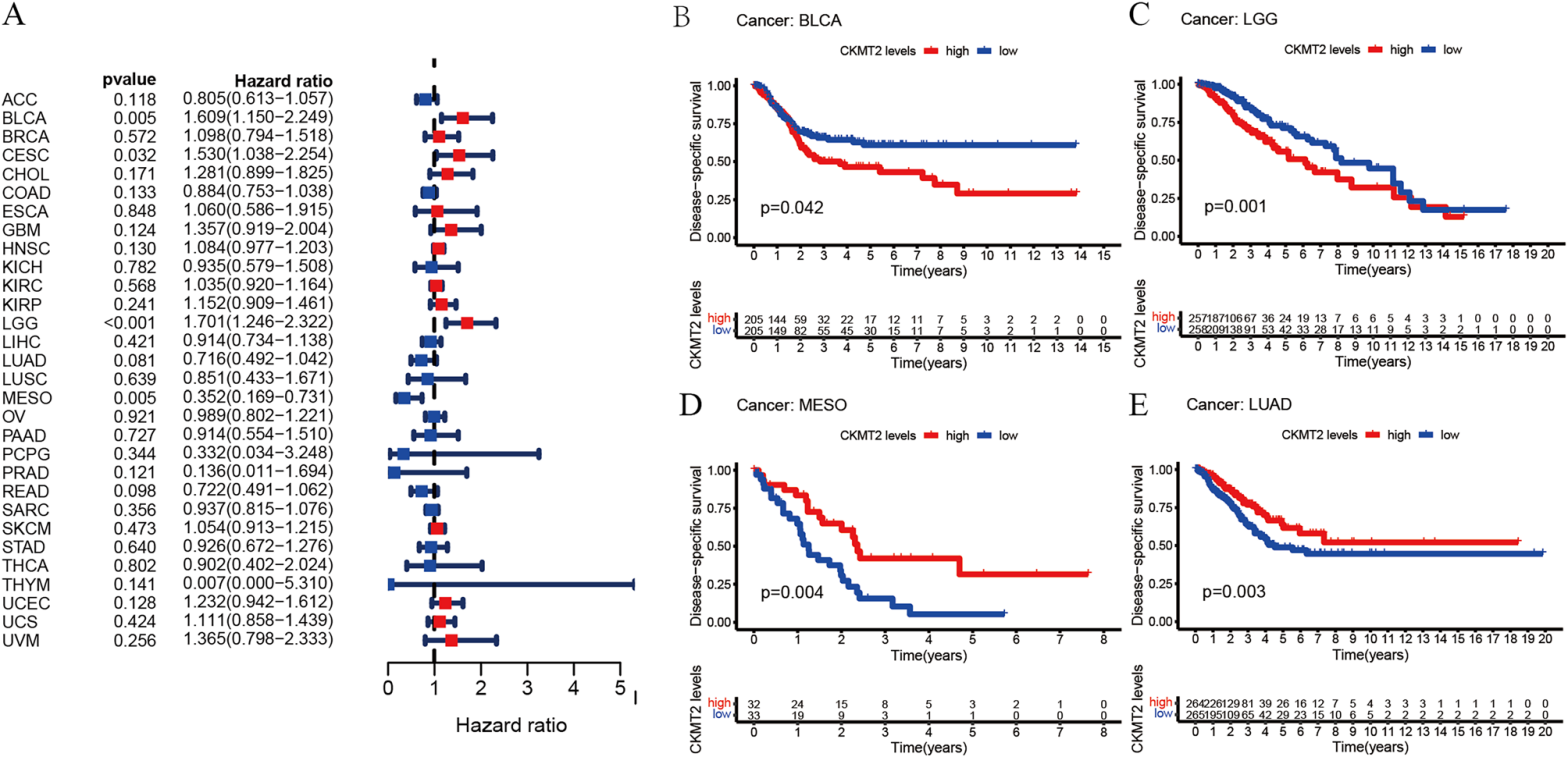
Relationship of CKMT2 expression with patient Disease-Specific Survival (DSS). (**A**) The forest map shows the univariate Cox regression analysis results for CKMT2 in TCGA pan-cancer samples. (**B**–**E**) Kaplan–Meier DSS curves of CKMT2 expression in the four most significantly associated tumors.

**Figure 6:**
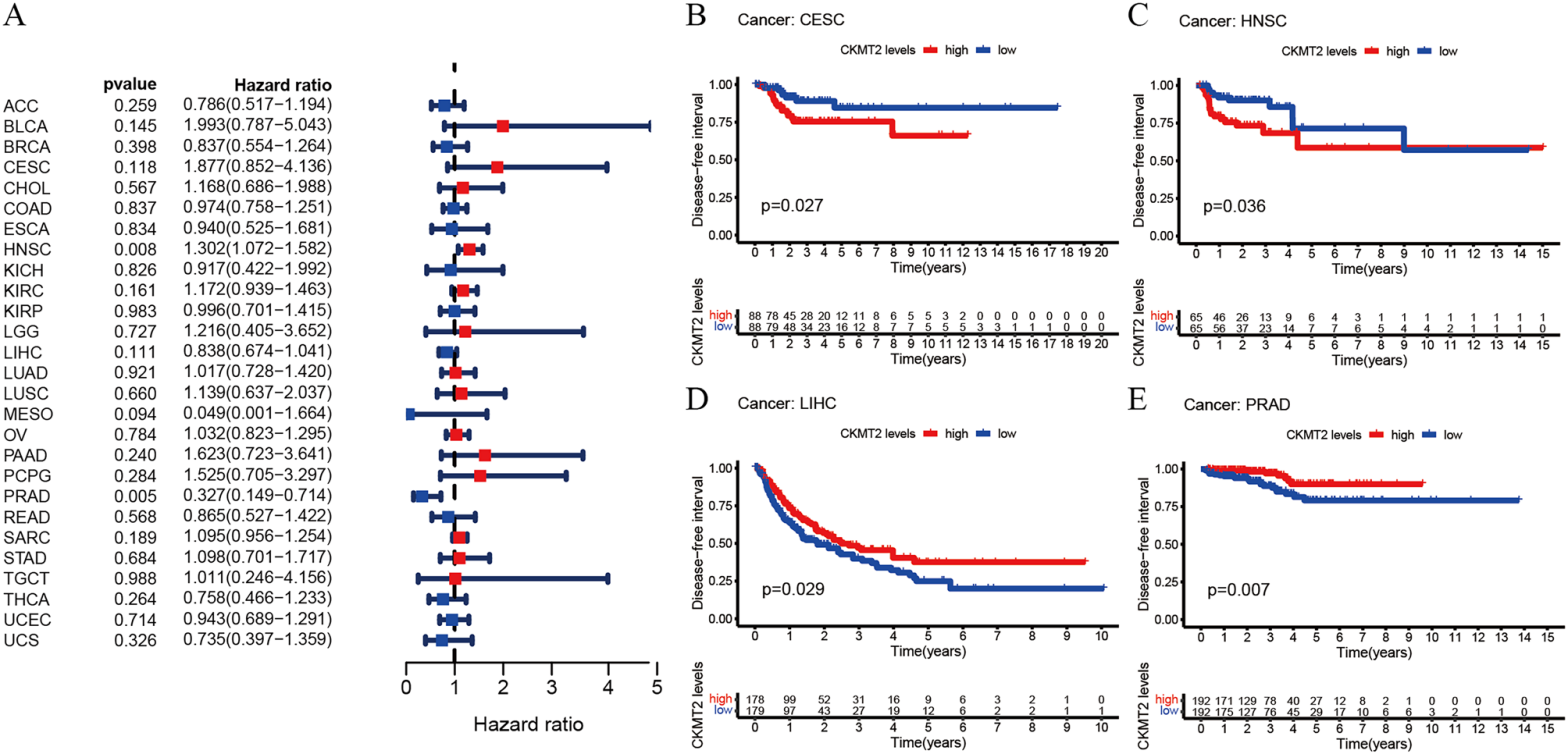
Relationship of CKMT2 expression with patient Disease-Free Interval (DFI). (**A**) The forest map shows the univariate Cox regression analysis results for CKMT2 in TCGA pan-cancer samples. (**B**–**E**) Kaplan–Meier DFI curves of CKMT2 expression in the most significantly associated tumors.

**Figure 7:**
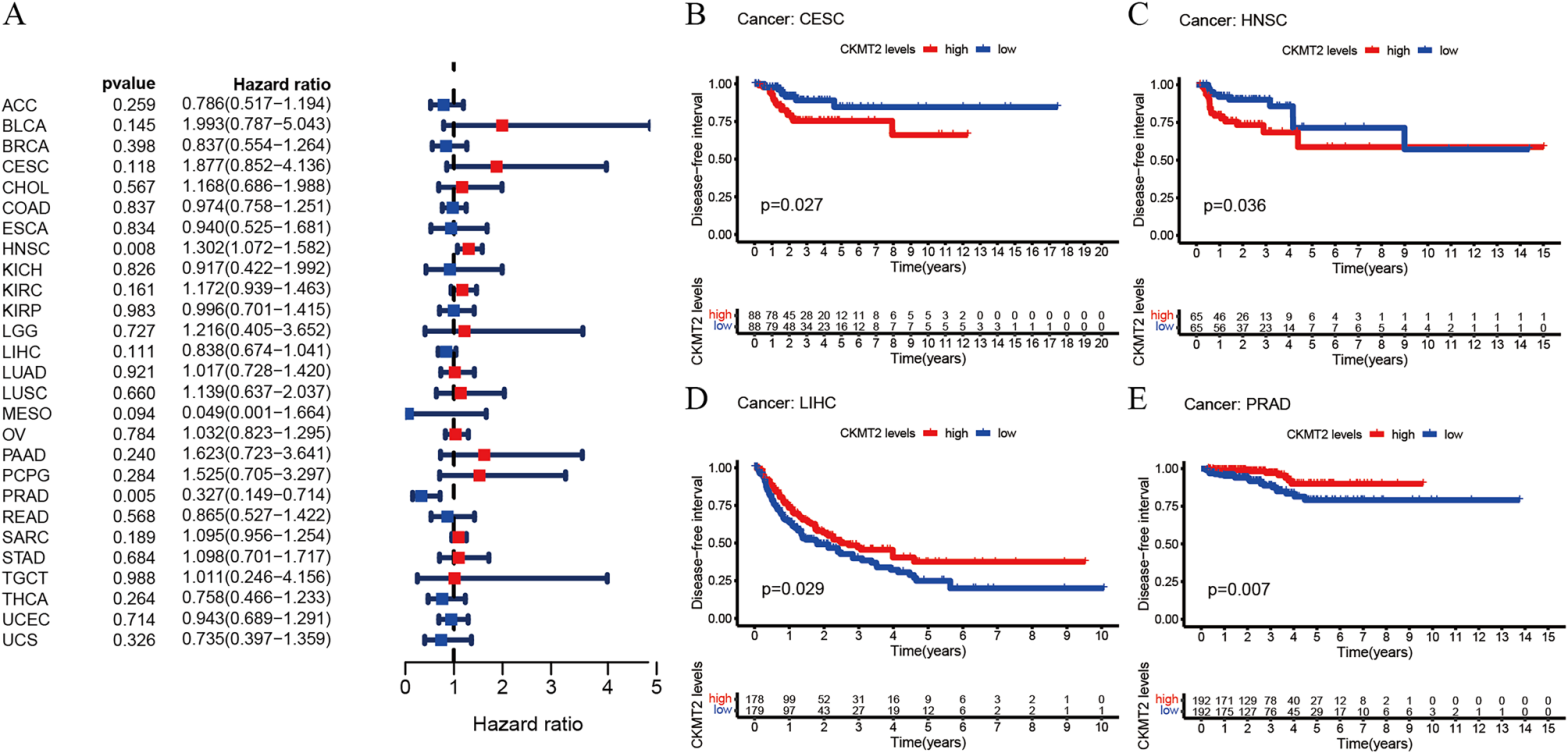
Relationship of CKMT2 expression with patient Progression-Free Interval (PFI). (**A**) The forest map shows the univariate Cox regression analysis results for CKMT2 in TCGA pan-cancer samples. (**B**–**D**) Kaplan–Meier PFI curves of CKMT2 expression in the three most significantly associated tumors.

The association between CKMT2 expression and cancer survival outcomes was investigated using both one-way Cox and KM analyses for OS, DSS, DFI, and PFI. Univariate Cox regression analysis of OS showed that CKMT2 overexpression was a risk factor for patients with BLCA, LGG, and THYM (HR > 1), whereas CKMT2 overexpression was a protective factor for patients with LAML, MESO, and READ (HR < 1) (Fig. 4A–E). KM analysis of OS showed that CKMT2 overexpression in BLCA, LGG had a poor prognosis, and CKMT2 overexpression in MESO, READ had a better prognosis. Cox regression analysis of DSS identified CKMT2 overexpression as a risk factor for BLCA, LGG, and CESC (HR > 1), but acted as a protective factor in MESO (HR < 1). KM analysis of DSS showed a poor prognosis of DSS in patients with higher CKMT2 expression in BLCA and LGG, but opposite results in LUAD and MESO (Fig. 5A–E). Cox regression analysis of DFI showed that CKMT2 overexpression was a risk factor for HNSC (HR > 1), but a protective factor for PRAD (HR < 1). In contrast, KM analysis of DFI showed a poor prognosis of DFI in patients with higher CKMT2 expression in CESC and HNSC, but a better prognosis in LIHC and PRAD (Fig. 6A–E). Finally, Cox regression analysis of PFI showed that CKMT2 overexpression was a risk factor for CESC and LGG patients (HR > 1) but a protective factor for MESO and PRAD patients (HR < 1). KM analysis of PFI showed that in CESC, CKMT2 overexpression had a poor prognosis, while in MESO and PRAD it had a better prognosis (Fig. 7A–D).

## Differential expression lncRNA analysis

**Figure 8:**
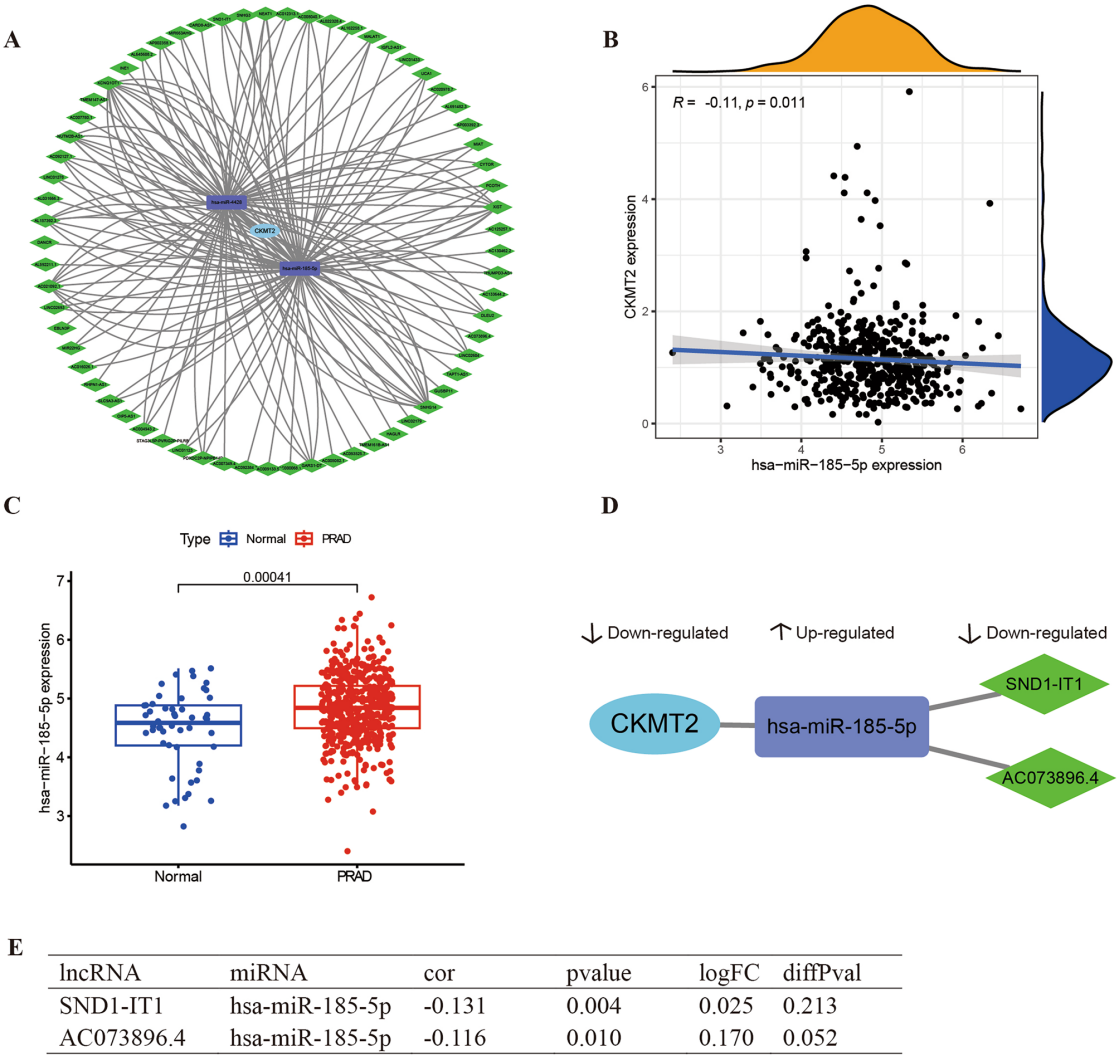
Recognition of two potential lncRNA-miRNA-mRNA ceRNA regulatory axes. (**A**) LncRNA-miRNA-mRNA network in PRAD. The blue nodes indicate CKMT2, the purple nodes indicate miRNA, and the green nodes indicate lncRNA. (**C**) The miRNA has-miR-185-5p was more highly expressed in PRAD than that in Normal. (**B**–**E**) The targeted regulation relationship among the lncRNA, miRNA, and mRNA of the two potential ceRNA regulatory axes in the PRAD development.

The corresponding lncRNAs for the hub miRNAs were predicted using ENCORI software^26^. The selection condition is set to (CLIP data ≥ 2). Two miRNAs were predicted to interact with CKMT2, hsa-miR-185-5p and hsa-miR-4428, respectively. The downstream lncRNAs of the two miRNAs were predicted simultaneously, including 49 lncRNA targets of hsa-miR-185-5p and 38 lncRNA targets of hsa-miR-4428. The number of CLIP sites and target sequences in each miRNA-mRNA, and lncRNA-miRNA relationship in the ENCORI database are shown in Supplementary Table S1. The lncRNA-miRNA-mRNA ceRNA network was constructed with Cytoscape. From the survival analysis of CKMT2 and PRAD, it is clear that CKMT2 is a protective factor for PRAD, so we chose to analyze the lncRNA-miRNA-mRNA regulatory axis of CKMT2 in PRAD. We constructed a network of CKMT2-associated ceRNAs in PRAD by target-pairing model genes in the ENCORI database (Fig. 8A). We extracted the expression data of differentially expressed lncRNAs, miRNAs, and CKMT2 from the PRAD dataset stored in The Cancer Genome Atlas (TCGA) database (Fig. 8 B,C). From the analysis, it can be concluded that the downregulation of lncRNA (SND1-IT1, AC073896.4) attenuated the regulation of miRNA (has-miR-185-5p). Expression of miRNA (has-miR-185-5p) was upregulated thereby downregulating CKMT2 expression (Fig. 8 D,E).

## The expression of CKMT2 is related to immune genes

**Figure 9:**
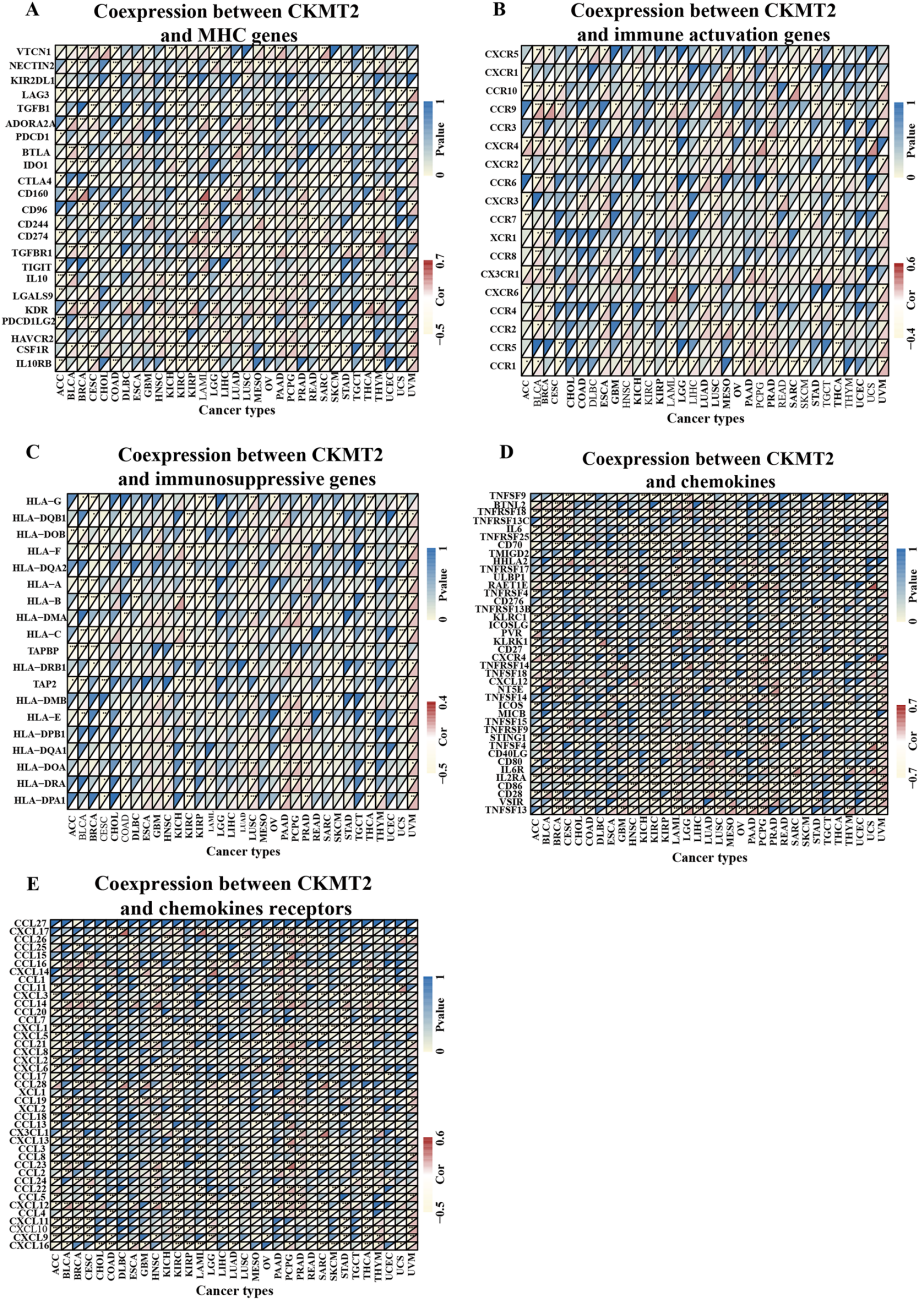
Co-expression of CKMT2 and immune-related genes. Co-expression of CKMT2 with (**A**) MHC gene, (**B**) immune activating gene, (**C**) immune suppressor gene, (**D**) chemokine gene, and (**E**) chemokine receptor gene. * *p* < 0.05, **p < 0.01, ***p < 0.001, ****p < 0.0001.

We carried out gene co-expression analysis to explore the relationship between CKMT2 expression and immune-related genes in different types of cancer. The genes encoding MHC, immune activation, immunosuppression, chemokine, and chemokine receptor proteins were analyzed (Fig. 9A–E). The results showed that CKMT2 was strongly associated with most immune-related genes in specific cancer types, such as ACC, BLCA, BRCA, KIRC, LUAD, PRAD, and THCA. Specifically, chemokine receptors such as CCL28, CCL16, CCL14, and CXCL12, and chemokines such as RAETIE, TNFSF13, BTNL2, and CXCR4 were positively correlated with the expression of CKMT2 in various tumors. MHC gene is co-expressed with CKMT2 in almost all cancer types, especially in BLCA, CESC, KIRC, LUAD, and THCA. In addition, immune activation genes and immunosuppressive genes are also closely related to the expression of CKMT2 in TCGA pan-cancer. In summary, these results suggest that the expression of CKMT2 is closely related to the biological functions of various immune-related genes and cytokines.

## CKMT2 is mainly enriched in tumor immunomodulatory pathways

**Figure 10:**
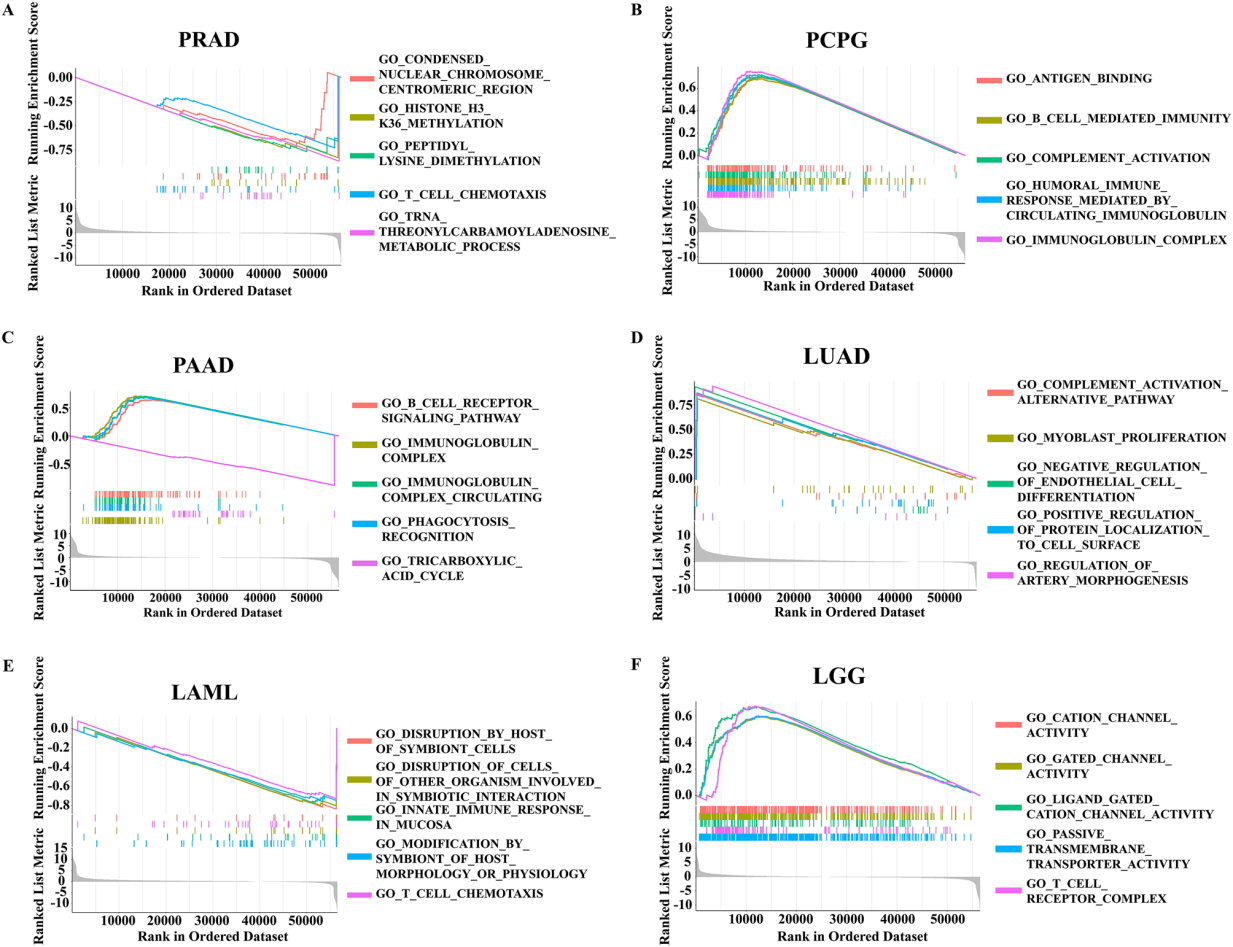
GO pathway analysis of CKMT2 in GSEA in pan-carcinoma. (**A**) PRAD; (**B**) PCPG; (**C**) PAAD; (**D**) LUAD; (**E**) LAML; (**F**) LGG.

To study the biological function of CKMT2 expression in different tumor tissues, the possible pathways of CKMT2 involved in GSEA in 33 tumor types of TCGA were evaluated. The results showed that CKMT2 was involved in tumor immunomodulatory pathways, especially the adaptive immune system, innate immune system, complement activation alternative pathway, and immunomodulatory interaction of immunoglobulin (Fig. 10A–F). These results suggest that CKMT2 plays an important role in regulating tumor immune metabolism and tumor immune microenvironment.

## The expression of CKMT2 is related to the tumor microenvironment

**Figure 11:**
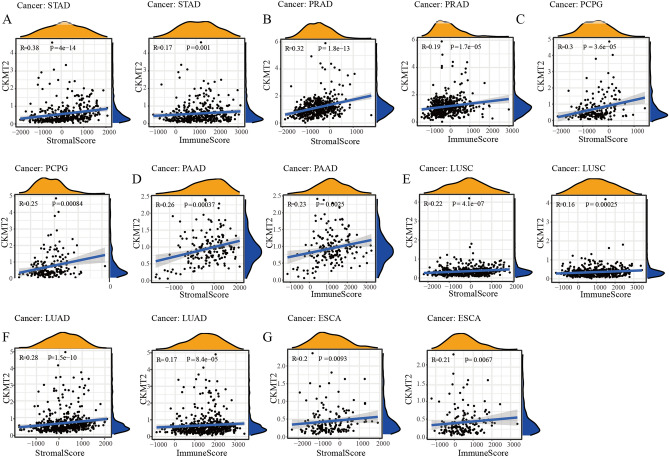
Relationship between CKMT2 expression and matrix score and immune score in seven cancers. CKMT2 expression is associated with (**A**) STAD, (**B**) PRAD, (**C**) PCPG, (**D**) PAAD, (**E**) LUSC, (**F**) LUAD, and (**G**) ESCA Stromal Score is positively correlated with Immune Score.

The tumor microenvironment (TME), as the living place of tumor cells, plays an important role in a variety of drug resistance and contributes to the progression and metastasis of cancer cells. Therefore, we used the ESTIMATE algorithm to calculate the immune score and matrix score respectively to study the correlation between CKMT2 expression and tumor microenvironment composition. ESTIMATE algorithm can estimate the matrix fraction (stromal score) and immune score (immune score) of tumor samples based on the expression data, which can be used to represent the existence of matrix and immune cells. The estimated score is obtained by adding the two fractions, which are used to estimate the tumor purity^27,28^. It can be concluded that the higher the content of stromal cells and immune cells, the lower the purity of tumor cells. The expression of CKMT2 was positively correlated with the immune scores of ESCA, LUAD, LUSC, PAAD, PCPG, PRAD, and STAD, indicating that the higher the content of immune cells, the higher the expression of CKMT2. The immune scores of CESC, KIRC, LAML, LGG, SARC, SKCM, THCA, and THYM were negatively correlated. In addition, the expression of CKMT2 was positively correlated with the matrix score of 11 cancers, indicating that the higher the content of stromal cells, the higher the expression of CKMT2, including BLCA, BRCA, ESCA, HNSC, LIHC, LUAD, LUSC, PAAD, PCPG, PRAD, and STAD. However, it was negatively correlated with LAML, OV, SARC, SKCM, and THCA. From the above data, it can be concluded that the seven cancer types that are positively correlated with the expression of TME and CKMT2 are STAD, PRAD, PCPG, PAAD, LUSC, LUAD, and ESCA (Fig. 11A–G).

## The expression of CKMT2 is associated with tumor-associated macrophage infiltration

To study the relationship between immune cell infiltration and CKMT2 expression at the pan-cancer level, we downloaded immune cell infiltration data from the database for correlation analysis. Based on the published data, we used the “CIBERSOFT” algorithm^29^ to evaluate 22 kinds of immune cells. In general, the expression of CKMT2 was positively correlated with the infiltration of many kinds of immune cells (Supplementary Table 2), including dendritic cells, monocytes, immature B cells, resting mast cells, macrophages M1 and NK cells, but negatively correlated with follicular helper T cells, macrophages M0, M2 and neutrophils (Supplementary Table 1). Interestingly, the expression of CKMT2 was correlated with different subsets of macrophages, positively correlated with the level of M1 macrophages, and negatively correlated with M0 and M2 macrophages. These results suggest that CKMT2 may help to increase the infiltration level of TAM, which may explain its tumorigenicity in most tumor types.

## Lower expression of CKMT2 can achieve better immunotherapy results

**Figure 12:**
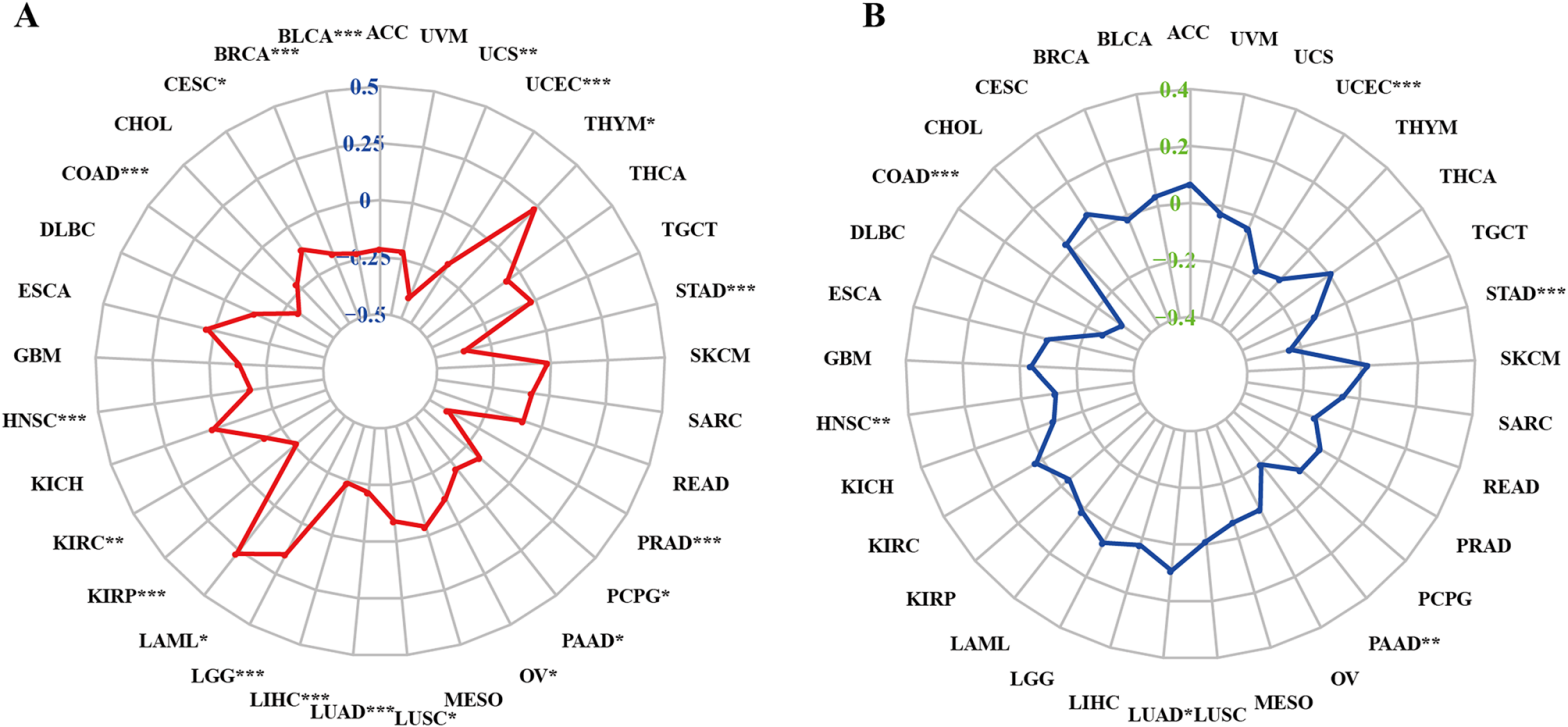
Relationship between CKMT2 expression and tumor mutation burden (TMB) and microsatellite instability (MSI). (**A**) TMB. (**B**) MSI. Spearman correlation test,*p < 0.05, **p < 0.01, ***p < 0.001, ****p < 0.0001.

Tumor mutation load (TMB) and Microsatellite instability (MSI) can predict the immunotherapy response of different tumor types. TMB is defined as the total number of somatic-coded mutations in a particular cancer, which is closely related to the effectiveness of immunotherapy in different types of human cancers^30^. MSI is a disease characterized by mononucleotide and oligonucleotide (short tandem repeat) repeats that reflect DNA mismatch repair (MMR) defects^31^. Similarly, MSI is a sign of a good response to immunotherapy. Many studies have shown that patients with high TMB/MSI increase response rates and show better results from immunotherapy. We evaluated the relationship between each of them and the expression of CKMT2 in pan-cancer. The correlation between CKMT2 expression and TMB was significant in all 17 kinds of cancer. The expression of CKMT2 in BLCA, BRCA, COAD, HNSC, KIRC, KIRP, LIHC, LUAD, LUSC, OV, PAAD, PCPG, PRAD, STAD, UCEC, UCS was negatively correlated with TMB. It was positively correlated with TMB only in LGG (Fig. 12A). Similarly, we further found that the expression of CKMT2 was negatively correlated with MSI in six cancers, including COAD, HNSC, PAAD, STAD, and UCEC, and positively correlated with MSI only in LUAD (Fig. 12B). From the above data, it can be concluded that the low expression of CKMT2 in COAD, HNSC, PAAD, STAD, and UCEC may have better immunotherapy results.

## Discussion

Mitochondrial creatine kinase (CKMT2) is one of the isoenzymes of creatine kinase (CK), which is closely coupled with adenosine triphosphate (ATP) output through adenine nucleotide transporters or carriers, making it an important participant in ATP synthesis and respiratory chain activities^7^. In the study of breast cancer cells, the researchers found that activating Y153 phosphorylation through the HER2/ABL axis stabilizes other mitochondrial CK isozymes CKMT1, thereby increasing phosphocreatine energy shuttling and promoting proliferation^32^. Although CKMT2 plays an important role in metabolism, the correlation of CKMT2 function in immune-oncology is still unknown. Here, we analyzed the expression profile and prognostic value of CKMT2 in pan-cancer by bioinformatics and explored its potential role in tumor immunology.

We first used TCGA data sets to evaluate the expression and prognostic significance of CKMT2 in pan-cancer. The results showed that compared with paracancerous tissues and normal tissues, CKMT2 gene mRNA was overexpressed in CKMT2 in 4 kinds of cancers. In contrast, low CKMT2 expression was observed in 14 cancers, and the IHC analysis of HPA was consistent with the expression of CKMT2 mRNA. CKMT2 is a key regulatory factor in the occurrence and development of osteosarcoma and is significantly related to OS in patients^13^. However, CKMT2 has not been widely studied in the field of cancer.

In this study, based on Kaplan–Meier and univariate Cox regression analysis, we also found that the up-regulated expression of CKMT2 was associated with poor prognosis, especially in patients with BLCA, LGG, KIRP, CESC, HNSC, and THYM. However, the high expression of CKMT2 is associated with a better prognosis in patients with PRAD, which means that the function of CKMT2 is more like a protective role in particular cancer. These findings suggest that CKMT2 is a potential biomarker for predicting the prognosis of pan-cancer.

Another important finding of this study is that CKMT2 plays an important role in tumor immunity. In the past few years, more and more studies have shown that the immune status of tumors is closely dependent on the composition and infiltration concentration of cells in their corresponding environment^33,34^. ESTIMATE algorithm has been proven to be convenient and quick to predict tumor purity, which reflects the characteristics of TME, and has been proven to be a prognostic factor for human malignant tumors, especially colon cancer patients^35^. Using the TCGA cohort, the expression of CKMT2 was positively correlated with the immune score of 7 cancers and negatively correlated with the immune score of 8 cancers. In addition, the expression of CKMT2 was positively correlated with the matrix score of 11 cancers and negatively correlated with 5 cancers. Furthermore, GSEA analysis indicated that CKMT2 was significantly associated with immune function, especially Humoral immunity. In addition, we found that CKMT2 was positively correlated with the infiltration level of many kinds of immune cells, including dendritic cells, monocytes, immature B cells, resting mast cells, macrophage M1, and NK cells. More importantly, we observed a significant correlation between CKMT2 expression and tumor-associated macrophages (TAM). TAM is the most abundant tumor-infiltrating immune cell group in TME, which usually differentiates into two opposite subtypes, namely the classical activated M1 subtype and the alternately activated M2 subtype^36^. Compared with the former, tumor-infiltrating M2 macrophages are closely related to the poor clinical prognosis of many malignant tumors^37^. More and more studies have shown that strategies targeting TAM can reduce the number of inhibitory macrophages in tumors, which can be used to enhance the efficacy of immune checkpoint blocking^38^. Based on these data, CKMT2 may have direct or indirect effects on macrophage polarization and subsequent immunosuppressive response. In addition, we further found that CKMT2 is co-expressed with genes encoding MHC, immune activation, immunosuppression, chemokine, and chemokine receptor proteins. All these findings suggest that the expression of CKMT2 is closely related to the immune infiltration of tumor cells, thus affecting the prognosis of patients.

TMB reflects the antigenic load in the tumor, so it is closely related to the efficacy of immune therapy. Previous studies have shown that high TMB is associated with better clinical outcomes of immune checkpoint inhibitors (ICI) in patients with melanoma^39^ and urothelial cancer^40^. In addition, TMB can be used as a prognostic and predictive biomarker of human cancer immunotherapy response. MSI is also a key biomarker of ICI reaction. The Food and Drug Administration (FDA) has approved high microsatellite instability (MSI-H) or mismatch repair defects (dMMR) as predictive biomarkers to guide the clinical application of ICI in some cancers^41^. In this study, we demonstrated that CKMT2 expression is associated with TMB in 17 cancers and MSI in 6 cancers. Therefore, CKMT2 can be used as a potential predictor of immunotherapy efficacy in these types of cancer.

However, we must admit some limitations in the current research. First of all, although we have integrated and analyzed information from multiple databases, these data sets are grouped without evaluating heterogeneity, which may reduce the reliability of our research results. Secondly, this study verified the expression of CKMT2 in colon and rectal cancer only at the protein level. Next, we will supplement experiments to clarify the mechanisms of CKMT2 in different types of cancers at the cellular and molecular levels. Although CKMT2 expression is associated with immune cell infiltration and patient survival in cancer, we cannot confirm whether CKMT2 may affect patient survival through immune pathways.

To sum up, our study systematically demonstrated the expression and prognostic value of CKMT2 in a series of cancers. The abnormal expression of CKMT2 is associated with poor prognosis of many types of cancer and with immune infiltration in TME, especially with TAM. In addition, CKMT2 expression is associated with TMB and MSI in many cancer types, suggesting that CKMT2 is associated with current predictors of ICI efficacy. However, these results are based on different data analyses, and future prospective experimental studies are needed to prove the specific role of CKMT2 in malignant tumors.

### Supplementary Information

Supplementary Information 1.

Supplementary Information 2.

## Data Availability

The datasets used and/or analyzed during the current study are available from the corresponding author upon reasonable request.
