# Supplementary material for: Prognostic value of mitochondrial CKMT2 in Pan-cancer and its tumor immune correlation analysis
Source: Sci Rep. 2024 Jan 3;14:342. doi: 10.1038/s41598-023-46468-3 (PMC10764887; doi:10.1038/s41598-023-46468-3)
Supplement: Supplementary file 1 — Supplementary Information 1. [file 41598_2023_46468_MOESM1_ESM.docx]

Supplementary Material

Prognostic value of mitochondrial CKMT2 in Pan-cancer and its tumor immune correlation analysis

**Wei Lin^1^, Jiamin Zhou^2^, Yili Ma^3^, Liuxing Ge^2^, Yiling Luo^2^, Yaobin Wang^2^, Sufang Zhou^1,2^***

*** Correspondence:** Sufang Zhou: [zsf200000@163.com](mailto:zsf200000@163.com)

^1^ State Key Laboratory of Targeting Oncology, National Center for International Research of Bio-targeting Theranostics, Guangxi Key Laboratory of Bio-targeting Theranostics, Collaborative Innovation Center for Targeting Tumor Diagnosis and Therapy, Guangxi Medical University, Nanning, Guangxi, 530021, China.

^2^ Department of Biochemistry and Molecular Biology, School of Basic Medical Sciences, Guangxi Medical University, Nanning, China

^3^Department of Pathology, Affiliated Cancer Hospital of Guangxi Medical University, Nanning, China

# Supplementary Figures and Tables

## Supplement
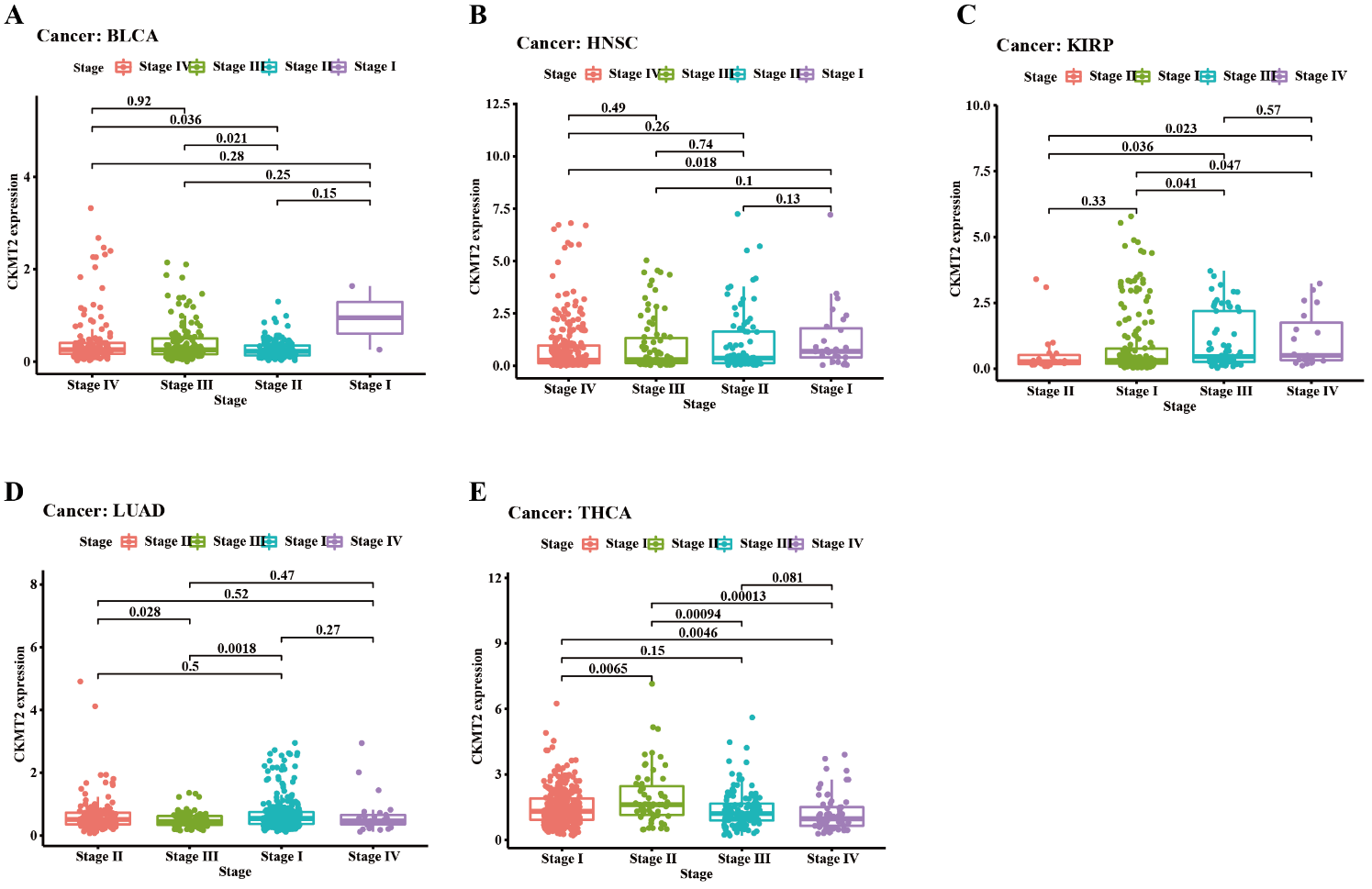


**Supplementary Figure 1.** Stage Ⅰ-Ⅳ CKMT2 expression in pan-carcinoma. (A-E) Differential expression of CKMT2 in different stages of pan-carcinoma.

## Supplementary Tables

Supplementary Tab.1 miRNA-mRNA, and lncRNA-miRNA relationship

| Gene | miRNA Name | Gene Name | Gene Type |
| --- | --- | --- | --- |
| CKMT2 | hsa-miR-185-5p | AC000068.1 | lncRNA |
| CKMT2 | hsa-miR-185-5p | AC005082.1 | lncRNA |
| CKMT2 | hsa-miR-185-5p | AC008040.1 | lncRNA |
| CKMT2 | hsa-miR-185-5p | AC009133.5 | lncRNA |
| CKMT2 | hsa-miR-185-5p | AC016026.1 | lncRNA |
| CKMT2 | hsa-miR-185-5p | AC020978.7 | lncRNA |
| CKMT2 | hsa-miR-185-5p | AC021092.1 | lncRNA |
| CKMT2 | hsa-miR-185-5p | AC073896.4 | lncRNA |
| CKMT2 | hsa-miR-185-5p | AC092127.1 | lncRNA |
| CKMT2 | hsa-miR-185-5p | AC092384.3 | lncRNA |
| CKMT2 | hsa-miR-185-5p | AC125257.1 | lncRNA |
| CKMT2 | hsa-miR-185-5p | AC130462.2 | lncRNA |
| CKMT2 | hsa-miR-185-5p | AL022328.4 | lncRNA |
| CKMT2 | hsa-miR-185-5p | AL031666.3 | lncRNA |
| CKMT2 | hsa-miR-185-5p | AL157392.3 | lncRNA |
| CKMT2 | hsa-miR-185-5p | AL592211.1 | lncRNA |
| CKMT2 | hsa-miR-185-5p | DANCR | lncRNA |
| CKMT2 | hsa-miR-185-5p | GUSBP11 | lncRNA |
| CKMT2 | hsa-miR-185-5p | HAGLR | lncRNA |
| CKMT2 | hsa-miR-185-5p | IGFL2-AS1 | lncRNA |
| CKMT2 | hsa-miR-185-5p | INE1 | lncRNA |
| CKMT2 | hsa-miR-185-5p | KCNQ1OT1 | lncRNA |
| CKMT2 | hsa-miR-185-5p | LINC01123 | lncRNA |
| CKMT2 | hsa-miR-185-5p | LINC01278 | lncRNA |
| CKMT2 | hsa-miR-185-5p | LINC02179 | lncRNA |
| CKMT2 | hsa-miR-185-5p | LINC02604 | lncRNA |
| CKMT2 | hsa-miR-185-5p | LINC02693 | lncRNA |
| CKMT2 | hsa-miR-185-5p | MALAT1 | lncRNA |
| CKMT2 | hsa-miR-185-5p | MIAT | lncRNA |
| CKMT2 | hsa-miR-185-5p | MIR663AHG | lncRNA |
| CKMT2 | hsa-miR-185-5p | NEAT1 | lncRNA |
| CKMT2 | hsa-miR-185-5p | NUTM2B-AS1 | lncRNA |
| CKMT2 | hsa-miR-185-5p | OIP5-AS1 | lncRNA |
| CKMT2 | hsa-miR-185-5p | PCOTH | lncRNA |
| CKMT2 | hsa-miR-185-5p | PDXDC2P-NPIPB14P | lncRNA |
| CKMT2 | hsa-miR-185-5p | SLC9A3-AS1 | lncRNA |
| CKMT2 | hsa-miR-185-5p | SND1-IT1 | lncRNA |
| CKMT2 | hsa-miR-185-5p | SNHG14 | lncRNA |
| CKMT2 | hsa-miR-185-5p | SNHG3 | lncRNA |
| CKMT2 | hsa-miR-185-5p | THUMPD3-AS1 | lncRNA |
| CKMT2 | hsa-miR-185-5p | TMEM147-AS1 | lncRNA |
| CKMT2 | hsa-miR-185-5p | UCA1 | lncRNA |
| CKMT2 | hsa-miR-185-5p | XIST | lncRNA |
| CKMT2 | hsa-miR-4428 | AC004943.2 | lncRNA |
| CKMT2 | hsa-miR-4428 | AC007349.4 | lncRNA |
| CKMT2 | hsa-miR-4428 | AC007780.1 | lncRNA |
| CKMT2 | hsa-miR-4428 | AC012313.1 | lncRNA |
| CKMT2 | hsa-miR-4428 | AC093525.7 | lncRNA |
| CKMT2 | hsa-miR-4428 | AC133644.2 | lncRNA |
| CKMT2 | hsa-miR-4428 | AL157392.3 | lncRNA |
| CKMT2 | hsa-miR-4428 | AL162258.1 | lncRNA |
| CKMT2 | hsa-miR-4428 | AL645608.2 | lncRNA |
| CKMT2 | hsa-miR-4428 | AL691482.3 | lncRNA |
| CKMT2 | hsa-miR-4428 | AP002358.1 | lncRNA |
| CKMT2 | hsa-miR-4428 | AP003392.3 | lncRNA |
| CKMT2 | hsa-miR-4428 | CARD8-AS1 | lncRNA |
| CKMT2 | hsa-miR-4428 | CYTOR | lncRNA |
| CKMT2 | hsa-miR-4428 | DLEU2 | lncRNA |
| CKMT2 | hsa-miR-4428 | EBLN3P | lncRNA |
| CKMT2 | hsa-miR-4428 | GARS1-DT | lncRNA |
| CKMT2 | hsa-miR-4428 | KCNQ1OT1 | lncRNA |
| CKMT2 | hsa-miR-4428 | LINC01433 | lncRNA |
| CKMT2 | hsa-miR-4428 | LINC02693 | lncRNA |
| CKMT2 | hsa-miR-4428 | MALAT1 | lncRNA |
| CKMT2 | hsa-miR-4428 | MIAT | lncRNA |
| CKMT2 | hsa-miR-4428 | MIR22HG | lncRNA |
| CKMT2 | hsa-miR-4428 | NEAT1 | lncRNA |
| CKMT2 | hsa-miR-4428 | NUTM2B-AS1 | lncRNA |
| CKMT2 | hsa-miR-4428 | PCOTH | lncRNA |
| CKMT2 | hsa-miR-4428 | PDXDC2P-NPIPB14P | lncRNA |
| CKMT2 | hsa-miR-4428 | RHPN1-AS1 | lncRNA |
| CKMT2 | hsa-miR-4428 | SNHG14 | lncRNA |
| CKMT2 | hsa-miR-4428 | SNHG3 | lncRNA |
| CKMT2 | hsa-miR-4428 | STAG3L5P-PVRIG2P-PILRB | lncRNA |
| CKMT2 | hsa-miR-4428 | TAPT1-AS1 | lncRNA |
| CKMT2 | hsa-miR-4428 | TMEM161B-AS1 | lncRNA |
| CKMT2 | hsa-miR-4428 | XIST | lncRNA |

Supplementary Tab.2 Correlation between CKMT2 expression and immune cells

| Cancer Type | CKMT2 expression | Immune cell type | R-value | P-value |
| --- | --- | --- | --- | --- |
| ACC | low | Dendritic cells activated | 0.49 | 0.00087 |
| ACC | low | Monocytes | 0.54 | 0.00026 |
| BLCA | low | B cells naive | 0.39 | 1.5e−13 |
| BLCA | low | Mast cells resting | 0.30 | 1.8e−08 |
| BRCA | low | B cells naive | 0.30 | < 2.2e−16 |
| CESC | low | B cells naive | 0.31 | 3.9e−08 |
| CESC | low | T cells CD4 memory activated | − 0.30 | 1.6e−07 |
| CHOL | high | NK cells activated | 0.60 | 0.00029 |
| COAD | high | T cells CD4 memory activated | 0.29 | 4.3e−10 |
| ESCA | low | B cells naive | 0.28 | 5e−04 |
| ESCA | low | Macrophages M0 | − 0.28 | 0.00055 |
| ESCA | low | Mast cells resting | 0.28 | 7e−04 |
| KIRP | low | B cells naive | 0.28 | 4.1e−06 |
| LAML | low | B cells naive | 0.37 | 2.7e−06 |
| LAML | low | Macrophages M2 | − 0.33 | 4.2e−05 |
| LAML | low | Neutrophils | −0.29 | 0.00025 |
| LAML | low | T cells CD4 naive | 0.29 | 0.00036 |
| LGG | low | Macrophages M1 | 0.30 | 4e−09 |
| READ | high | T cells CD4 memory activated | 0.29 | 3e−04 |
| SARC | low | Mast cells resting | 0.28 | 1.3e−05 |
| SARC | low | T cells CD4 memory activated | − 0.26 | 5.2e−05 |
| STAD | low | B cells naive | 0.31 | 1.6e−09 |
| STAD | low | Macrophages M0 | − 0.27 | 9.3e−08 |
| STAD | low | Mast cells resting | 0.29 | 9.8e−09 |
| STAD | low | T cells CD4 memory activated | − 0.28 | 4.6e−08 |
| TGCT | low | B cells naive | 0.29 | 0.00026 |
| THCA | low | Dendritic cells resting | − 0.26 | 1.7e−07 |
| THYM | low | Mast cells resting | 0.30 | 0.00093 |
| UVM | low | Macrophages M1 | 0.56 | 0.00031 |
